# Delayed Cranial Nerve Palsies and Chiari Type I Malformation After Epidural Anesthesia in the Setting of Childbirth

**DOI:** 10.7759/cureus.12871

**Published:** 2021-01-23

**Authors:** James P Caruso, Salah G Aoun, Jean-Luc K Kabangu, Olutoyosi Ogunkua, Carlos A Bagley

**Affiliations:** 1 Neurosurgery, University of Texas Southwestern Medical Center, Dallas, USA; 2 Anesthesiology, University of Texas Southwestern Medical Center, Dallas, USA

**Keywords:** epidural, childbirth, chiari, cranial nerve palsy, analgesia

## Abstract

Epidural analgesia is an efficient method of controlling pain and has a wide spectrum of therapeutic and diagnostic applications. Potential complications may occur in a delayed fashion, can remain undiagnosed, and can be a source of significant morbidity. We present a 37-year-old woman presented with severe spontaneous occipital headaches, diplopia, and dizziness that occurred spontaneously six weeks after giving birth. Her primary method of pain control during labor was epidural analgesia. Her neurologic exam revealed a cranial nerve six palsy with ptosis, and her brain MRI demonstrated a Chiari I malformation which had not been previously diagnosed. CT myelography of the lumbar spine revealed extradural contrast extravasation within the interspinous soft tissue at L1-L2, which was the site of her prior epidural procedure. She underwent epidural blood patch administration, and her cranial nerve palsy resolved along with all of her other symptoms. The development of concurrent Chiari I malformation and cranial nerve palsy after epidural anesthesia is an exceptionally rare occurrence. Neurologic complications after epidural anesthesia are likely under-reported, since patients are often lost to follow-up or have subtle neurologic signs which can easily be missed. This frequently delayed presentation emphasizes the importance of patient education and the necessity of a detailed neurological exam when symptoms occur.

## Introduction

Epidural anesthesia has a variety of therapeutic and diagnostic applications, and it is a well-tolerated procedure with minimal risk of significant complications. Among the most common side effects is post-dural puncture headache (PDPH) [1], which is reported in up to 2.6% of cases and can occur if cerebrospinal fluid (CSF) is accidentally released [1,2]. More severe neurologic complications, including cranial nerve (CN) palsies and loss of function, are less common. Current evidence suggests that cranial nerve palsies can result from CSF egress after accidental dural puncture, which leads to intracranial hypotension and subsequent brain sag and nerve dysfunction due to traction [3-6]. Quincke et al. reported the first case of CN VI palsy following dural puncture over 100 years ago [7], and multiple publications have drawn attention to this complication as a consequence of epidural anesthesia [8]. Chiari I malformation following spinal fluid release is also thought to result from negative intracranial pressure and brain sag, and it can occur with persistent CSF leak [9,10]. However, reports of this phenomenon occurring secondary to epidural anesthesia are exceedingly rare, and no reports exist that describe the simultaneous occurrence of cranial nerve palsy and Chiari malformation after epidural anesthesia. We present the first documented case of symptomatic Chiari I malformation and new cranial nerve palsy occurring six weeks after epidural anesthesia and resolving completely after treatment. We also provide a review of the literature to facilitate the understanding, diagnosis, and management of these complications.

## Case presentation

The patient is a 37-year-old woman who presented with a chief complaint of six weeks of occipital headaches, persistent dizziness, and diplopia. Her medical history was notable for childbirth three months earlier via vaginal delivery without immediate complications. Epidural anesthesia was used for pain control during labor. Of note, no accidental dural puncture was suspected at the time of procedure, and cerebrospinal fluid leak was not observed at the procedure site. Six weeks following delivery, she began to experience intermittent occipital headaches. The headaches did not appear to be postural in nature, and she had no similar episodes in the past. After developing dizziness and diplopia, she was evaluated by a neuro-ophthalmologist and an MRI was obtained. She was diagnosed with a Chiari I malformation and CN VI palsy. Additionally, MRV did not demonstrate any evidence of venous sinus thrombosis. An eye patch provided temporary relief of diplopia, and she was referred to a general neurosurgery clinic for further evaluation. One week later, her neurologic exam was significant for the progression of her symptoms, a left CN VI palsy, and near-complete ptosis of the left eye which suggested a new CN III injury. She demonstrated no overt pupillary dysfunction, did not demonstrate papilledema, her remaining cranial nerves were intact, and her strength and sensory function were not affected.

**Figure 1:**
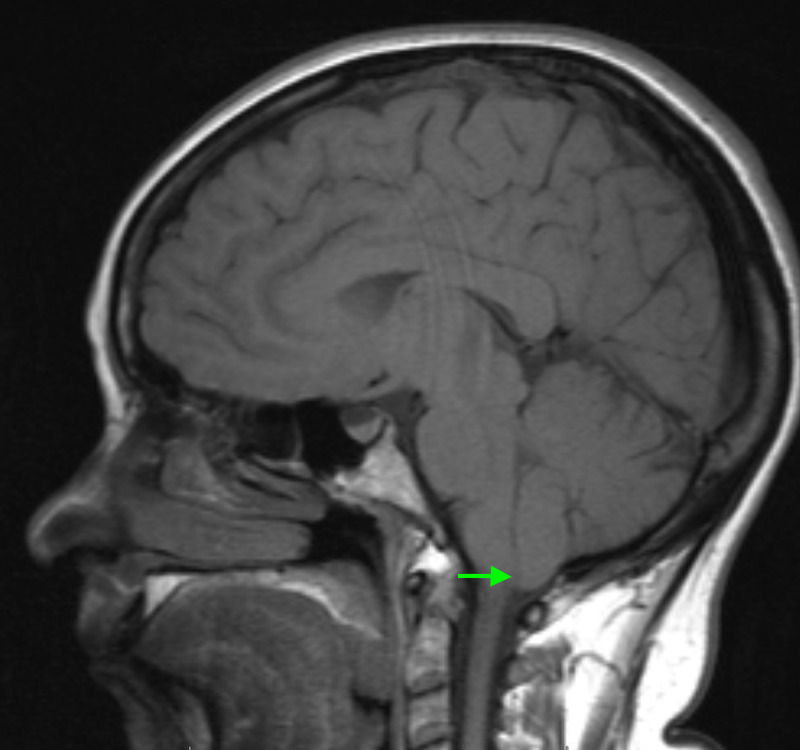
T1 sagittal MRI revealing a Chiari I malformation with approximately 5-6 mm of tonsillar herniation and no upper cervical syrinx.

**Figure 2:**
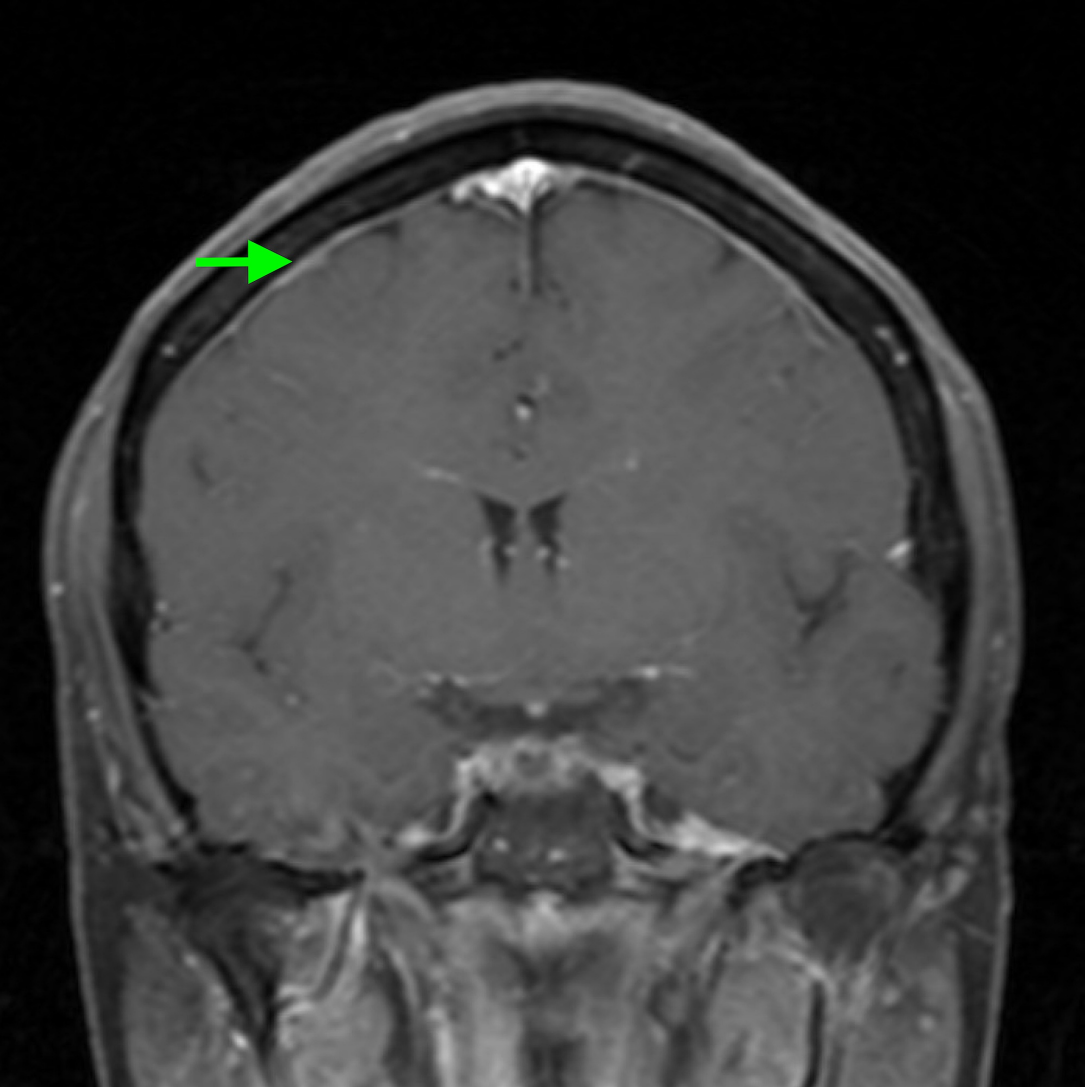
T1 coronal post-contrast MRI revealing enhancement of the convexity dura.

**Figure 3:**
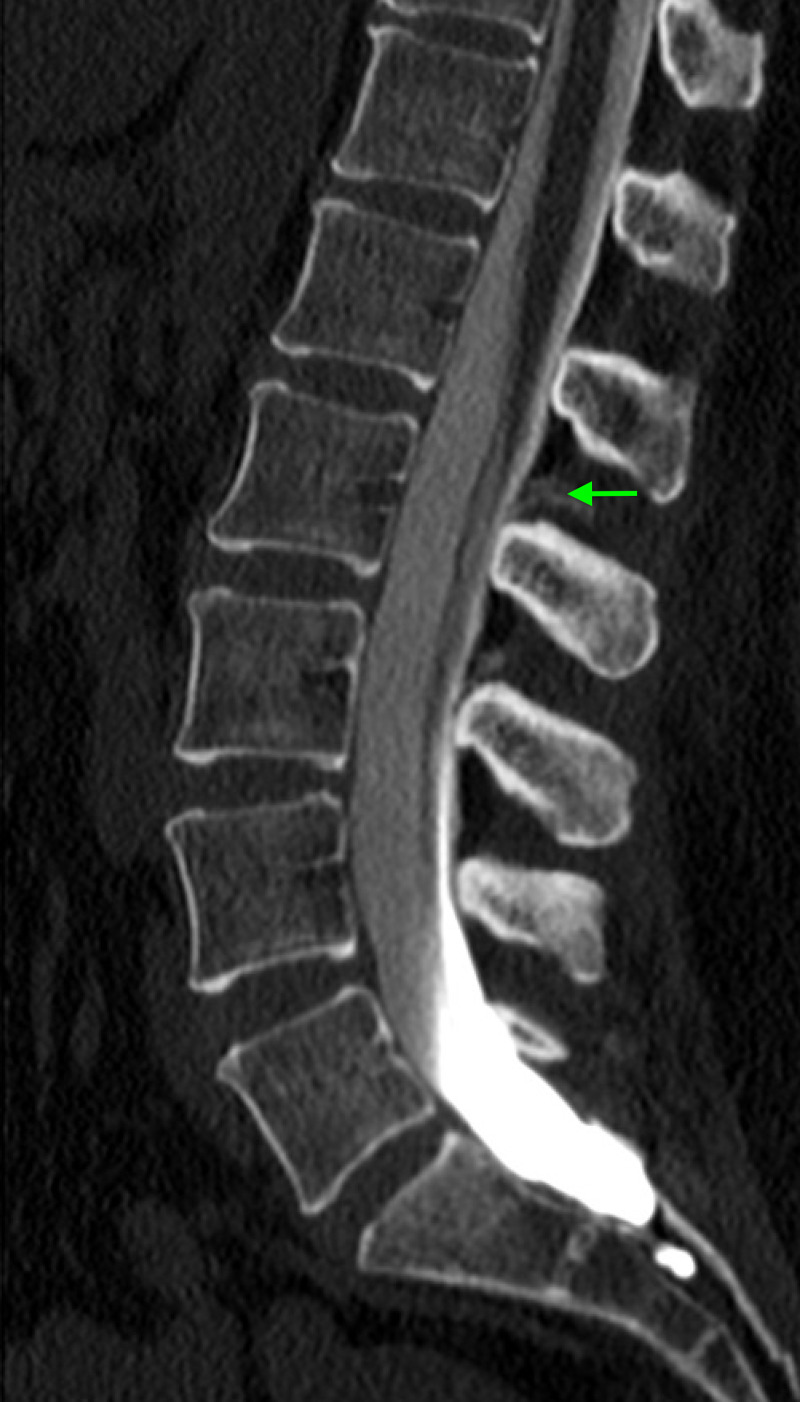
CT myelogram of the lumbar spine revealing extradural contrast extravasation within the interspinous soft tissue at L1-L2 with additional spread between the right L2-3 and L3-4 nerve roots.

Her MRI revealed a Chiari type I malformation with approximately 5-6 mm of tonsillar herniation and no upper cervical syrinx (Figure 1). The post-contrast MRI sequences revealed enhancement of the convexity dura, which can signify negative intracranial pressure, but did not demonstrate evidence of pituitary hyperemia, generalized brain sag, or subdural effusions (Figure 2). A CT myelogram of the lumbar spine was obtained, and it revealed extradural contrast extravasation within the interspinous soft tissue at L1-L2 with additional spread between the right L2-3 and L3-4 nerve roots (Figure 3). The myelogram used the L5-S1 interlaminar spaces for the injection of contrast material. These findings were consistent with a CSF leak originating an L1-2. She received an epidural blood patch at L1-2 and experienced almost immediate headache relief. At her one-month follow-up visit, her diplopia had improved, her CN VI palsy and ptosis had resolved, and her MRI showed stable tonsillar herniation with stable venous sinuses. At her three-month follow-up visit, her headaches, dizziness, and diplopia had completely disappeared, and she was able to resume her normal activities.

## Discussion

CN palsies as a consequence of epidural anesthesia are likely under-reported, since patients are often lost to follow-up if their symptoms are mild [4]. When patients do present for follow-up, they are rarely subjected to a detailed neurologic exam capable of detecting subtle changes in extra-ocular muscle function [4]. Chambers et al. performed a review of 43 case reports of CN palsies following central neuraxial block for obstetric applications [11]. Only four of these patients had bilateral CN deficits, and the most commonly affected CNs were CN VI and CN VII. The majority of CN palsies were preceded by PDPH [11], in a similar fashion to our case. However, only one patient developed multiple cranial nerve palsies after epidural anesthesia, and none of the reported patients developed concurrent Chiari I malformations. Nishio et al. provide a similar analysis of 95 cases of extraocular muscle paralysis after dural puncture procedures [12]. While an intradural block was the most common causative procedure, 11% of cases occurred after epidural anesthesia [12]. In these cases, it is likely that symptoms resulted from a dural breach causing ectopic CSF egress. They also note that CN VI palsies have the greatest likelihood of resolution with treatment [12]. While CN VI appears to be the most frequently affected nerve after epidural anesthesia in these series, other CN palsies are also reported. Multiple case reports describe the co-occurrence of CN V palsy and Horner’s syndrome following epidural anesthesia [13-16], with one case also affecting CN XII [15]. Epidural anesthesia is also rarely associated with more severe neurologic complications, including cortical vein thrombosis and subdural hematoma [17]. In our case, the patient presented from a left CN VI palsy and ipsilateral ptosis. Her ptosis may represent acute-onset Horner’s syndrome resulting from diffusion of the analgesic agent into the cervical sympathetic fibers innervating the face, but she did not have any other symptoms of Horner’s syndrome, such as myosis and enophthalmos. Additionally, her symptoms would have resolved over time with breakdown of the anesthetic agent. The delayed onset of her ptosis more likely suggests that she developed an incomplete CN III palsy, which may have worsened had she not sought treatment.

When CN complications arise as a result of epidural anesthesia, epidural blood patching has been described as the treatment of choice. Multiple case reports have outlined the association between epidural blood patch application and symptom resolution in the weeks to months following treatment [7,9]. Chambers and Bhatia’s analysis revealed that 17 patients with CN palsies were treated successfully with epidural blood patch, but they also suggest that CN palsies associated with frank CSF leak and severe intracranial hypotension may not respond to epidural blood patch [11]. Other accounts also suggest that CN palsies may remain refractory to treatment [11,12,18]. Palliative measures such as eye patches or prism glasses often help minimize patient discomfort associated with extraocular muscle impairment until more definitive treatment is provided [12].

Development of Chiari I malformation is even more uncommon than the development of cranial nerve palsy after epidural anesthesia, since brain sags significant enough to cause tonsillar herniation would require a large amount of iatrogenic CSF loss, which typically does not occur. We initially questioned whether the patient’s Chiari I malformation was a pre-existing condition, rather than a new finding, considering that her CT myelogram only demonstrated a minimal volume of CSF that tracked into the subcutaneous tissue. However, her imaging also demonstrated contrast-enhancement along the convexity dura, and her symptoms responded rapidly to epidural blood patch, which suggests that intracranial hypotension contributed to her symptoms. Atkinson et al. described seven cases of Chiari I malformation secondary to spontaneous spinal fluid leak leading to intracranial hypotension [9]. Presentations were heterogeneous in their series, and symptoms ranged from seven weeks to one year after treatment by epidural blood patch or direct surgical repair, which may imply a different pathophysiology compared to symptoms resulting from accidental iatrogenic durotomies during epidural anesthesia [19].

Neurologic complications of epidural anesthesia can present in a surreptitious fashion, and since deficits that last more than eight months may remain permanent [12], prompt identification is paramount. While no evidence-based guidelines exist to determine the appropriate interval for neurologic follow-up after epidural anesthesia, we recommend a two-week post-procedural follow-up that includes a detailed neurologic examination. We also recommend pre-procedural patient education in case neurological deterioration is delayed. While our case appeared to present in a delayed fashion, Chambers and Bhatia’s analysis suggests that >90% of CN palsies related to epidural or combined spinal-epidural anesthesia occurred within the first two weeks post-procedure [11]. Scheduled post-procedure follow-up would also provide an opportunity for clear physician-patient communication regarding warning signs of intracranial hypotension and cranial nerve injury, which would facilitate early diagnosis and treatment if the patient develops symptoms in a delayed fashion. 

## Conclusions

Development of concurrent Chiari malformation and CN palsy is an exceptionally rare consequence of epidural anesthesia, and we present the first documented case of this phenomenon. Our patient’s delayed presentation emphasizes the importance of diligent neurologic surveillance when administering epidural anesthesia. If patients present with positional headaches or cranial nerve symptoms after epidural anesthesia, providers should maintain a low threshold to evaluate for intracranial hypotension. If imaging and clinical findings are consistent with intracranial hypotension secondary to dural violation, then epidural blood patch should be discussed. Available literature does reflect the incidence and management options for cranial nerve complications following epidural anesthesia, but more investigation is necessary to determine appropriate diagnostic and treatment guidelines for post-epidural development of Chiari I malformation.
